# The Attitude of Insurance Committees Towards Medical Benefit

**Published:** 1913-03-01

**Authors:** 


### THE ATTITUDE OF INSURANCE COMMITTEES TOWARDS MEDICAL BENEFIT.

### The New Forms and Instructions.

From the very outset of the National Health Insurance Act it has been evident that its provisions would press hardly upon certain sections of the insured, unless the Commissioners and Committees remedied matters by special regulations. None too soon this has been recognised, and steps have now been taken to obviate some of the more glaring types of hardship. These steps have been signalised by the publication of sheaves of special forms, letters of instruction, and circumlocution generally. It is instructive to notice that some of them are quite simple and straightforward; others so highly complicated, indeed unintelligible to those who will have to use them, that they will inevitably become complete dead letters, useful only to the Committees as an excuse for doing nothing further to promote the real welfare of those to whom the Act is proving an incubus and an oppression.

### Concessions for Hospital Employees.

As these forms and the grievances which have elicited them touch several classes of worker whose claims to exceptional treatment have been advocated in The Hospital, it is worth while to examine them in detail.

To begin with, Form 43 (*b*) deals with the medical benefits of nurses, male attendants, and institutional employees of all kinds, to whose peculiar position under the Act as compared with that of ordinary wage-earners we have repeatedly drawn attention in these columns. Our readers are therefore familiar with the cogent reasons for granting special treatment to institutional servants; but a brief recapitulation may not be out of place. Nurses, porters, attendants, engineers, ward-maids, cooks, and all the varied personnel of a modern hospital or asylum have always received, by custom, free medical attendance when ill from the hospital staff—paid and honorary—and free accommodation in the wards or in special sick-rooms. (House surgeons, it may be remarked, have been excluded from the operation of the Act by the Commissioners, so their case does not need to be further considered.) This provision of medical attendance is, beyond all question, far superior to any medical benefit by the panel system which the Insurance Act can provide. So that the hospital employees who are insured persons have been up to now in the position of being made to pay for benefits much inferior to those they already get for nothing. Moreover, their treatment from their own institution included free drugs, dressings, and appliances, which those attended by panel doctors have to get from the chemists who accept the terms offered by the Act, or under its regulations.

To meet this very obvious injustice it is now provided that hospital employees may apply for permission to make their own arrangements with their institution for medical treatment and attendance (including medicines and appliances); and to facilitate the matter, each form of application contains space for the signatures of about sixty or seventy insured persons. The third sheet of this Form 43 (*b*) also contains an undertaking, to be signed by the chairman and secretary of the governing body, guaranteeing that the treatment shall be "not inferior" in nature, quality, and extent to that provided by the Insurance Committee, and that various other conditions will be duly complied with. When this form has been duly filled in, it remains only for the local Insurance Committee to give its assent to the proposal.

### The Financial Provisions.

So far, so good. What about the recompense to the hospitals for relieving the Committees of all liability for medical benefit in respect of their own employees? The rational plan is for the hospital to receive, in respect of every such employee, a money payment representing the whole of medical benefit, less the amount required for sanatorium purposes. That is to say, the hospital should receive the whole of what, under the panel system, goes to doctor and chemist, including the sixpence for the domiciliary treatment of tuberculosis. This, apparently, is the plan which has been adopted, though a certain ambiguous passage in the Medical Benefit Regulations is quoted on the back of the form which seems to imply that only some smaller sum might be available. Substantially, however, there is a good prospect of the arrangement proving fair and equitable, and the result will be that hospitals, now called upon to pay annually a thirteen shilling tax in respect of every insured employee, will receive back more than half of that sum without undertaking any liability greater than that which they have always voluntarily accepted hitherto. Not, perhaps, a bargain that a business man would be proud of, but, at any rate, better than their position under the Act hitherto.

### Commercial Travellers and Actors.

There are other large classes of the community for whom the panel system is to all intents valueless; such are actors, commercial travellers, and others who, by the nature of their occupation, never stay long in one place, and are frequently away from home for considerable periods. These classes, too, present claims for consideration so strong that they cannot be refused. If they were merely permitted to make their own arrangements for medical benefit, receiving each year for that purpose from the Committee of the district in which they live a sum representing the cost of medical benefit, it is held that no satisfactory assurance could be obtained by the Committee in advance that proper treatment would be made certain. Therefore, the contract system is in these cases recommended to be abandoned altogether, and a system of repayment to the insured person of sums expended by him on medical attendance and medicines to be adopted.

This involves complicated account keeping by the insured persons, on special forms—43 (*a*). It involves also that he himself is directly liable to the doctor who attends him and the chemist from whom he gets his medicine for their charges, and at the end of each year he will recover some part of the money thus actually expended. To begin with, he cannot send in an application for the actual amount of the bills he has had to pay, but only for the bills as they would have been if calculated on a scale laid down by the Insurance Committee.

Even when the Form 43 (*b*) is satisfactorily filled up, complete with doctors' signatures, particulars about night visits, special visits, fractures, anæsthetics, and many other details; and when the contribution is calculated which the insured may claim; even then, it does not follow that he will actually receive that sum. It may happen that the pool available for distribution that year will suffice for the payment of a percentage only of the cost of medical benefits administered in this way; and if so, the unfortunate insured will find himself in receipt of a percentage of a portion of his out-of-pocket expenditure on his health. What is to happen in years of low sickness-rate, when the pool may be more than enough to meet the calls upon it, is not vouchsafed; in fairness, any such surplus should be carried forward to the pool of the next year, when sickness may be rife, but the forms contain no provision that this shall be done.

### Free Choice of Doctor Abolished.

We have dealt so far with certain cases in which the Commissioners have actually intimated or plainly hinted that Committees will do well to grant permission to the insured to make arrangements outside the panel system. But it is quite clear from Memo. 143, lately issued, that the vast bulk of the insured are to be dragooned on to the panel system whether they like it or not. Under Section 15 (3) of the Act, it is open to Committees to grant applications of insured persons to make their own arrangements for medical benefit, and very large numbers of them who are dissatisfied with the prospect of panel treatment have sent in such applications. The Commissioners in this memorandum give a broad hint to the Committees to refuse these applications, or to make the process so difficult that the applicants will soon get discouraged. Thus, the free choice of doctor demanded by the British Medical Association and promised by the Government has already been abolished. For we have no hesitation in describing Form 43, taken in conjunction with Memo. 143, as being really an intricate but highly effective way of making the panel system compulsory for the insured. The Commissioners and the Committees are in effect treating it as a crime for an insured person to want to retain the services of his old-time doctor, or of any other medical man who has refused to join the panels. Domestic servants especially will be sufferers by this policy. There is a certain specious appearance of sympathy about the attitude of the Commissioners to this class; but of practical help almost none. The Committees are a good deal worse, for they make no pretence of trying to remedy the evil.

Let us note first what the Commissioners are doing for this large class, many of whom have been attended in the past by the family practitioner of the household in which they are employed. If this doctor happen not to be on the panel, they cannot obtain his services as part of medical benefit, except by being allowed to "make their own arrangements for medical benefit," which Section 15 (3) of the Act provides for. Many of them, accordingly, have sent in such applications, especially in districts where the panels are ill-staffed, both in quantity and quality. But to discourage applicants as far as possible beforehand, Form 43 is issued, which they must fill up before the application will be considered. This form seems to have been especially devised for the express purpose of preventing the insured from taking advantage of their rights under Section 15 (3). It bristles with long and technical clauses, and is full of questions concerning the private affairs of the applicant.

The only step which the Commissioners view with even modified approval for mitigating this very real grievance, is that of allowing practitioners not on the regular panel to go upon it solely for a limited number of their old patients or for servants in the houses where they attend the employers. But this they will only allow provided that all the other doctors on the panel agree to it, and provided that all other insured persons have been allotted to a doctor. These provisions make it very doubtful whether the concession is worth the paper it is written on.

Let us turn now to the Committees. At Dundee, for instance, the Committee has refused, though only by a small majority, to allow servants or anyone else to contract with doctors not on the panel; twelve hundred was the number of the applications sent in. At Yarmouth the same thing has happened, though the number of cases was very much smaller. The same is true of other districts, and particularly of London, where the number of those desirous of taking advantage of Section 15 (3) is probably greater than anywhere else. Lady St. Helier championed the servants on this latter Committee, but her proposal was defeated by a large majority. One speaker told Lady St. Helier that hers was an insidious attempt to favour a particular class. It is a little difficult to see anything insidious in a motion (at a meeting open for the first time to the press) which expressly mentions domestic servants as those upon whom the Committee should confer this favour. But the friendly society element upon these Committees is, as has been foreseen from the start, absolutely dominant; reason and justice are of little account in the scales against the preponderant majority on the Committees. And so the servants have got to change their doctor, because the Committees want to score off the British Medical Association.

